# Duodenal gangliocytic paraganglioma, a rare entity among GEP-NET: a case report with immunohistochemical and molecular study

**DOI:** 10.1186/1746-1596-9-54

**Published:** 2014-03-12

**Authors:** Fabiana Tatangelo, Monica Cantile, Alessia Pelella, Nunzia Simona Losito, Giosuè Scognamiglio, Franco Bianco, Andrea Belli, Gerardo Botti

**Affiliations:** 1 Pathology Division, Istituto Nazionale Tumori “Fondazione G Pascale”-IRCCS, Naples, Italy; 2 Division of Surgical Oncology, Istituto Nazionale Tumori “Fondazione G Pascale”-IRCCS, Naples, Italy

**Keywords:** Gangliocytic paraganglioma, ND markers, Neuro D1

## Abstract

Gastroenteropancreatic neuroendocrine tumors are the most incident neuroendocrine tumors. In the new WHO classification (2010) the embryological derivation of each neoplastic entity is one of the most important parameters. Gangliocytic Paraganglioma is a tumor originating in the hindgut, a rare neoplasm, generally affecting the second portion of the duodenum, the majority of which are benign.

Cases of gangliocytic paraganglioma with local metastasis or local recurrence have also been reported.

We describe a GP in a 48-year-old caucasian male with an unusual site (4th portion of duodenum) and an interesting immunohistochemical and molecular pattern. In particular, we examined the expression of some neuroendocrine markers and a marker of neuronal differentiation, NeuroD1, whose expression can help to better understand the nature of this neoplasia.

**Virtual slides:**

The virtual slides for this article can be found here: http://www.diagnosticpathology.diagnomx.eu/vs/3720959161096807

## Background

Gangliocytic paraganglioma (GP) is a rare gastrointestinal tumor of uncertain histogenesis, with immunohistochemical features of neuroendocrine type.

This neoplasia, in the context of the new WHO classification (2010) of Gastroenteropancreatic neuroendocrine (GEP-NET) tumors is classified as tumor derived from the hindgut [1].

Usually, it is located in the second part of the duodenum and, in a few cases, in the esophagus, jejunum, pylorus, pancreas and upper mediastinum and is characterized by benign clinical behavior and a favorable outcome.

However, recent studies have highlighted the presence of paraganglioma in other sites, particularly in the retroperitoneum [2], with a case that metastasizes to the vertebra [3].

Moreover, cases of regional lymph node metastasis and local recurrence have been reported, suggesting a potential malignant progression of this tumor [4,5].

Histologically GP is a triphasic neoplasia. It is composed of three cellular types: epithelioid, ganglion and spindle cells, whose identification is supported by immunohistochemical (IHC) detection of neuronal and neuroendocrine markers (synaptophysin, chromogranin A, CD56, S-100, NSE and Somatostatin) [6].

Currently, the only prognostic marker for neuroendocrine tumors is Ki67 index expression associated with mitotic index. Molecular markers indicative of poor prognosis have not yet been identified.

Recently, a new marker, NeuroD1 (Neurogenic Differentiation 1), has been shown to act as a neuronal differentiation factor. NeuroD1 is a transcription factor originally identified in beta pancreatic cells and it is able to convert epithelial cells into neurons in Xenopus Embryos [7]. Moreover, NeuroD1 has been identified as a new neuroendocrine marker in GEP-NET and prostate cancer (pCA). In GEP-NET its expression seems to be related to differentiation degree [8], while in pCA its expression is more frequent than Chromogranin A [9,10]. Cytoplasmic positive staining for NeuroD1 was also strongly associated with MPI (microscopic perineural invasion).

In this report we present a case of Gangliocytic paraganglioma on which some neuroendocrine markers (Syn, CgA, CD56, NSE) and NeuroD1 expression have been tested by IHC. On the same sample NeuroD1 expression has also been determined by quantitative Real Time PCR.

## Case presentation

GP shows evidence of neuronal differentiation, also supported by cell positivity for several neuroendocrine (ND) markers.

In this case, a 48-year-old caucasian man was referred to our Institution after recurrent upper GI bleeding with melena. The patient reported a four month history of intermittent coffee ground stools, fatigue and weight loss. Clinical examination was uneventful and the patient was hemodinamically stable with 8.4 g/dl value of Haemoglobin and a Hematocrit of 28%. Iron deficiency was also present. No history of previous pathologies was reported. Pan-colonoscopy was negative and a standard gastroscopy had been performed at another Institution, revealing a 4 cm bulky mass of the distal duodenum. Gastroscopy was repeated in our institution with pediatric instrument to have a better evaluation of the mass. The lesion, located in the 4th duodenal portion, was included in the duodenal wall and covered with normal mucosa with a bleeding surface. Multiple biopsies were performed but the results were inconclusive.

A CT-scan was also performed, revealing a thickening of the 4th portion of the duodenal wall and non-lymphadenopathies.

The patient underwent an exploratory laparotomy with segmental resection of the 4th portion of the duodenum and the first jejunal loop. An end to end anastomosis between the 4th duodenal portion and the jejunum was carried out. The patient was discharged on the 6th post-operative day after an uneventful course.

Twenty-four months after surgery, the patient was diagnosed disease free.

Macroscopically the surgical specimen showed a polypoid lesion, covered with smooth mucosa with microerosion area, of 4 cm × 4 cm × 3 cm. All tumor samples were stained with H/E (hematoxilin/eosin). IHC stains were then performed.

**Figure 1:**
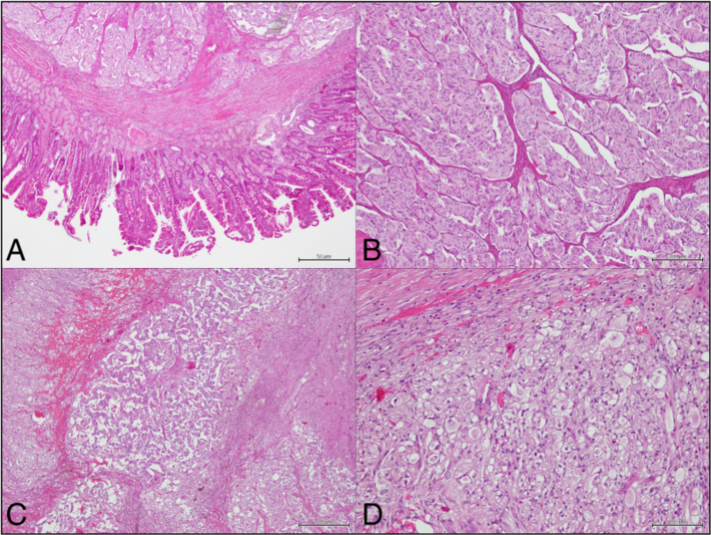
H&E: A) panoramic view with epithelioid and ganglion cells (4X); B) high magnification of epithelioid cellular elements (20X); C) panoramic view with epithelioid, spindle and ganglion cells (10X); D) high magnification of ganglion cells (20X).

Under microscopic observation the tumor was composed of epithelioid cell nests, areas of spindle cells and scattered ganglion cells (Figure 1). The neoplastic proliferation affected submucosal layers up to the lamina propria and was covered with duodenal mucosa devoid of morphological abnormalities, with focal areas of erosion of the epithelial layer.

**Figure 2:**
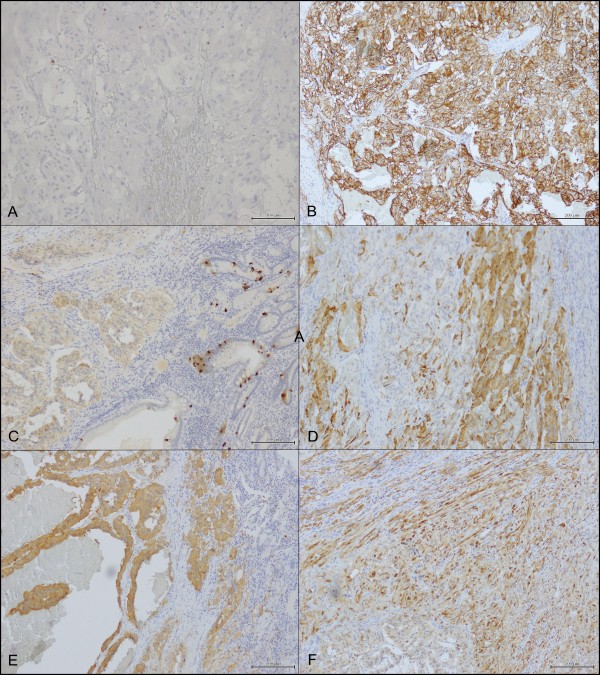
Immunostaining: A) Low expression (< 2%) of ki67 (10X); B) High expression of CD56 in epithelioid cells (10X); C) low expression of CgA (10X); D) Low/Moderate expression of Somatostatin (20X); E) High expression of Synaptophysin (10X); F) High expression of S100 protein in spindle cells (10X).

The panel of immunohistochemical markers used showed a negative cellular reaction for cytokeratin (CK) 7 (Dako, OVTL 12/30, 1: 200), CK20 (Dako, Ks20.8, 1:50), CDX2 (Dako, DAK-*CDX2*, 1:50) and CD117 (Dako, CD117, 1:50). A low positive cellular reaction was present for Vimentin (NeoMarkers, Westinghouse, California, D9, 1:1000.), S100 (*Dako*, 1:3000), Chromogranin A (Dako, A0430, 1:1500 ***)*** and Somatostatin (Abcam, ab103790, 1:200) while a strong positive cellular reaction was detected for Synaptophysin (Dako,1:3000), CD56 (Dako, 123C3, 1:50) and NSE (Dako, BBS/NC/VI-H14, 1:100) (Figure 2).

The final histological examination revealed a GP tumor.

After histological diagnosis, we evaluated the expression by IHC of NeuroD1 marker with primary antibody against human NeuroD1(Abcam, ab60704,1:200) for one hour of incubation. The sections were rinsed in TBS and incubated for 20 min with Novocastra Biotinylated Secondary Antibody (RE7103), a biotin-coniugated secondary antibody formulation that recognize mouse and rabbit immunoglobulins.

The sections were then rinsed in TBS and incubated for 20 min with Novocastra Streptavidin-HRP (RE104). Peroxidase reactivity was visualized using a 3,3′-diaminobenzidine (DAB).

IHC staining of Neuro D1 was scored as follows: - , if < 5% of tumor cells were stained; +, if there were focal, weak tumor cells staining; ++, if there were moderate tumor cells staining; +++, if there were marked tumor cells staining.

**Figure 3:**
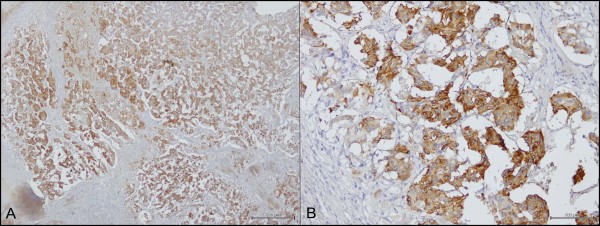
Neuro D1 immunostaining: A) cytoplasmic positivity in epithelioid and ganglion cells (4X); B) cytoplasmic positivity in epithelioid cells (40X).

NeuroD1 showed a marked positivity (+++), (Figure 3) mostly cytoplasmic, especially in ganglion and epithelioid cells.

Finally, for gene expression analysis, total RNA was isolated from a frozen biopsy of the tumor stored in our Institutional Bio-Bank, using RNeasy Mini Kit (Qiagen GmbH, Hilden, Germany) following the manufacturer’s instructions. The sample was treated with RNase-free DNase (Qiagen GmbH, Hilde, Germany) to prevent amplification of genomic DNA. A total of 1 mg RNA was subjected to cDNA synthesis. Quantitative Real Time -PCR (qRT PCR) was performed in a Light Cycler system (Roche Molecular Biochemicals, Mannheim, Germany) using specific TaqMan Gene Expression Assays for human NeuroD1 (Real Time Designer Assay cod. 05583055001, Roche Molecular Biochemicals).

**Figure 4:**
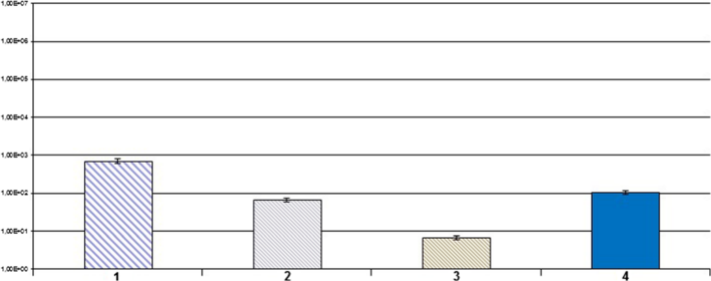
**Real time PCR expression of NeuroD1: 1) pancreatic normal sample; 2) pancreatic tumor sample; 3) prostatic tumor sample; 4) GP sample.** All reactions were performed in triplicate and data are expressed as mean of relative amount of mRNAs levels.

qRT PCR analysis showed NeuroD1 over-expression compared to other cancer samples used as controls (Figure 4).

## Discussion

GP is a rare neuroendocrine tumor of the digestive tract (GEP-NET ), originating in the hindgut, and mainly located at the second portion of the duodenum. It is usually benign.

Histopathological diagnosis of the disease requires the detection of a triphasic pattern of growth, represented by three cellular components: epithelioid cells, spindle shaped cells and ganglion-like cells [1].

The majority of the reported cases of duodenal GP has been of benign nature, even if some cases in literature showed the presence of regional lymph nodes metastasis [5]. Distant metastases have not been observed. Radical surgery with lymph node dissection is therefore required.

In the new WHO classification of NET(2010), the only parameter of prognostic value is represented by Ki67 index expression.

The case reported in this paper was characterized by a sessile polypoid lesion that at routine histopathological examination was diagnosed as duodenal GP, with negativity for CK 7 and CK 20 and with positivity for several neuroendocrine markers.

Since the identification of new molecular markers of both diagnostic and prognostic value becoming increasingly necessary, we decided to evaluate the expression of another neuroendocrine marker, NeuroD1, recently described in GEP-NET and other malignancies.

Our data, derived from immunohistochemistry, confirmed the positive protein expression of the NeuroD1 marker in this case of GP. Furthermore, we have also determined NeuroD1 gene expression on the same fresh cryopreserved sample, showing even in this case an overexpression of the marker.

The aberrant expression of NeuroD1 in GP, although a very rare tumor, could complete the panel of neuroendocrine markers for the diagnosis of these tumors.

Moreover, NeuroD1 may be identified on a largest casuistry of NETs to verify its role in the pathogenesis of these malignancies.

## Conclusions

Even if GP follows a benign course and invasive growth patterns and lymph node metastasis are rare events, the identification of new molecular markers markers associated with its neuroendocrine differentiation, is necessary.

In this context, Neuro D1 could be an important element also for the development of more tailored therapies for patients with this disease, as already described for other neuroendocrine tumors [11-13].

## Consent

Written informed consent was obtained from the patient for publication of this manuscript and any accompanying images. A copy of the written consent is available for review by the Editor-in-Chief of this journal.

## Competing interests

The authors declare that they have no competing interests.

## Authors’ contributions

FT and MC were responsible for the conception and design of the study. FB and AB were responsible for provision of study materials or patient. GS and AP collected and assembled data and samples for immunohistochemical analysis. MC and AP were responsible for the Real Time PCR analysis. FB, NSL and GB were responsible for immunohistochemical evalutation. All authors were involved in manuscript writing and provided final approval of the manuscript.
